# High Temperature and Bacteriophages Can Indirectly Select for Bacterial Pathogenicity in Environmental Reservoirs

**DOI:** 10.1371/journal.pone.0017651

**Published:** 2011-03-15

**Authors:** Ville-Petri Friman, Teppo Hiltunen, Matti Jalasvuori, Carita Lindstedt, Elina Laanto, Anni-Maria Örmälä, Jouni Laakso, Johanna Mappes, Jaana K. H. Bamford

**Affiliations:** 1 Centre of Excellence in Evolutionary Research, Department of Biological and Environmental Science, University of Jyväskylä, Jyväskylä, Finland; 2 Centre of Excellence in Virus Research, Department of Biological and Environmental Science and Nanoscience Centre, University of Jyväskylä, Jyväskylä, Finland; 3 Department of Biosciences, University of Helsinki, Helsinki, Finland; University of Liverpool, United Kingdom

## Abstract

The coincidental evolution hypothesis predicts that traits connected to bacterial pathogenicity could be indirectly selected outside the host as a correlated response to abiotic environmental conditions or different biotic species interactions. To investigate this, an opportunistic bacterial pathogen, *Serratia marcescens*, was cultured in the absence and presence of the lytic bacteriophage PPV (*Podoviridae*) at 25°C and 37°C for four weeks (N = 5). At the end, we measured changes in bacterial phage-resistance and potential virulence traits, and determined the pathogenicity of all bacterial selection lines in the *Parasemia plantaginis* insect model *in vivo*. Selection at 37°C increased bacterial motility and pathogenicity but only in the absence of phages. Exposure to phages increased the phage-resistance of bacteria, and this was costly in terms of decreased maximum population size in the absence of phages. However, this small-magnitude growth cost was not greater with bacteria that had evolved in high temperature regime, and no trade-off was found between phage-resistance and growth rate. As a result, phages constrained the evolution of a temperature-mediated increase in bacterial pathogenicity presumably by preferably infecting the highly motile and virulent bacteria. In more general perspective, our results suggest that the traits connected to bacterial pathogenicity could be indirectly selected as a correlated response by abiotic and biotic factors in environmental reservoirs.

## Introduction

Virulence, i.e. the decrease in host fitness caused by its associated parasite [1], is traditionally thought to coevolve in reciprocal selection between the host and the parasite. While this might be the case with obligate pathogens, many opportunistic pathogens reside most of their life cycle in environmental reservoirs without ever facing their potential hosts. This raises a question: could some other factors indirectly select the pathogenicity of opportunistic pathogens as a correlated response in the absence of suitable hosts? The coincidental evolution hypothesis [1]–[3] suggests that bacterial pathogenicity could be a by-product of selection acting on a parasite's life-history traits that increase its fitness in environmental reservoirs and which coincidentally also affects its virulence in hosts. For example, the competitive and cooperative abilities of bacteria have probably originally evolved to increase bacterial fitness and survival in natural microbial communities. However, these same traits also affect the severity of an infection by affecting how fast bacteria can proliferate within their hosts [4], [5]. Similarly, protozoan predation has been shown to increase bacterial pathogenicity because the same traits that originally evolved as defence mechanisms against protists can be used to invade the hosts, or after successful colonization, evade the host immune system [6]–[11]. Yet, even though previous studies have documented a positive correlation between bacterial virulence and survival in environmental reservoirs, the disease outbreaks driven by opportunistic bacterial pathogens are relatively rare. This suggests that some selective forces in environmental reservoirs are also likely select for lowered bacterial pathogenicity.

Bacteria constantly encounter numerous enemies in microbial communities. For example, ubiquitous bacteriophages, i.e. parasitic viruses that replicate within bacterial cells, can effectively constrain bacterial survival in nature [12]. A wide array of laboratory experiments has shown that bacteriophages can drive rapid bacterial evolution by imposing strong selection for phage-resistant bacteria [13]–[16]. Furthermore, phage-resistance has been shown to correlate negatively with some other bacterial life-history traits, such as growth efficiency [13], [17]–[19] and motility [20]–[22], which both are important traits for bacterial pathogenicity. Motility, for example, helps pathogens colonise suitable niches within the host [23], [24], while growth efficiency can determine how fast bacteria can exploit their hosts [4], [25], [26]. Thus, if phage-resistance leads to trade-off with bacterial virulence factors phages could potentially select for lowered bacterial pathogenicity in environmental reservoirs [13], [17]–[21]. This hypothesis is supported by several studies where phage-resistance has been shown to correlate with lowered pathogenicity in *Serratia marcescens* [27], *Bacillus thuringiensis* [20] and *Salmonella enterica* serovar Entereditis [28], however there is yet no direct experimental evidence demonstrating that phages can select for lowered bacterial pathogenicity through trade-offs with bacterial virulence factors.

It is also possible that abiotic environmental conditions can drive evolutionary changes in bacterial virulence. For example, selectively neutral environmental conditions can lead to loss of virulence genes through random drift [29] when pathogens are released from their hosts to environmental reservoirs [29] or cultured in novel medium in the absence of their hosts [30]. However, it is possible that some environmental factors increase bacterial pathogenicity. For example, temperature is known to be important environmental factor for the expression of several bacterial virulence factors [31]. It has been shown previously that elevating the growth temperature increases the pathogenicity of *Shigella* species [32] and *Legionella pneumophila* bacteria [33]. Furthermore, high temperatures can partially explain the evolution of more virulent strains of *Flavobacterim columnare* in fish farms [34] and drive the pathogenesis of marine macroalgae infecting *Ruegeria* sp. bacteria [35]. Even though high temperature-mediated increase in bacterial virulence could be phenotypically reversible and down regulated in lower temperatures [32], [33], it is possible that some changes could be genetic. For example, it has been shown that bacterial adaptation to high temperature environment improves its competitive ability also in lower temperature regimes [36], [37]. As a result, high temperature environments could indirectly select for more pathogenic bacterial pathogens at both phenotypic and genotypic level. However, there is as yet no experimental evidence how biotic and abiotic environmental factors interact in shaping bacterial pathogenicity in environmental reservoirs.

In this study, we investigated how parasitic phages and thermal environment affect the evolution of bacterial pathogenicity traits *in vitro*, and how these changes correlate with bacterial virulence *in vivo*. Based on the literature, we hypothesized that bacteriophages could select for lowered bacterial pathogenicity if the phage-resistance is traded off with bacterial virulence traits e.g. growth efficiency [13], [17]–[19]. In contrast, high environmental temperature could select for increased bacterial pathogenicity if it upregulates bacterial virulence traits [32]–[33], [38], or if high temperature environments selects for bacterial genotypes with improved competitive (and host exploitation) ability [36], [37]. It is also possible that phage and temperature treatments interact. As a result, selection by phages could have different effects for the bacterial virulence depending on the temperature environment. To study these hypotheses, we performed a microcosm experiment (see Figure S1 for methods diagram) where the opportunistic bacterial pathogen, *Serratia marcescens* (known to be able to infect plants, nematodes, insects, fishes and mammals [39]), was cultured either in the presence or absence of the parasitic lytic bacteriophage, PPV (*Podoviridae*), in both low (25°C) and high (37°C) temperature regimes for four weeks. At the end of the experiment, bacteria were isolated from all the microcosms to determine changes in several potential virulence related traits including growth efficiency, motility, biofilm formation [8] and resistance against the ancestral phage. All bacterial traits were measured at both 25°C and 37°C temperatures. Bacterial growth efficiency can determine the severity of infection through competitive and social interactions between different bacterial genotypes [4], [25], [26], [40]. Motility is known to correlate with the level of bacterial pathogenicity [24], [41], while it can also affect phage-susceptibility if the pilus or flagellar expression affects phage attachment efficiency [22], [42]–[44]. Bacterial biofilm formation plays an important role in many chronic infections and the bacterial biofilms has been observed to evolve resistant against phages [45]. Insect hosts are widely used to distinguish virulent clinical and non-virulent environmental strains of opportunistic bacteria [46]–[48]. Thus, changes in bacterial pathogenicity were compared *in vivo* in wood tiger moth (*Parasemia plantaginis*, Arctiidae) model host, which has been previously shown to vary in its resistance against different *S. marcescens* bacterial strains [40]. The survival assays were conducted only at 25°C: a temperature, which is more close to the natural maximum temperatures of the host species (originally isolated from Finland, see Materials and Methods).

## Results

**Figure 1:**
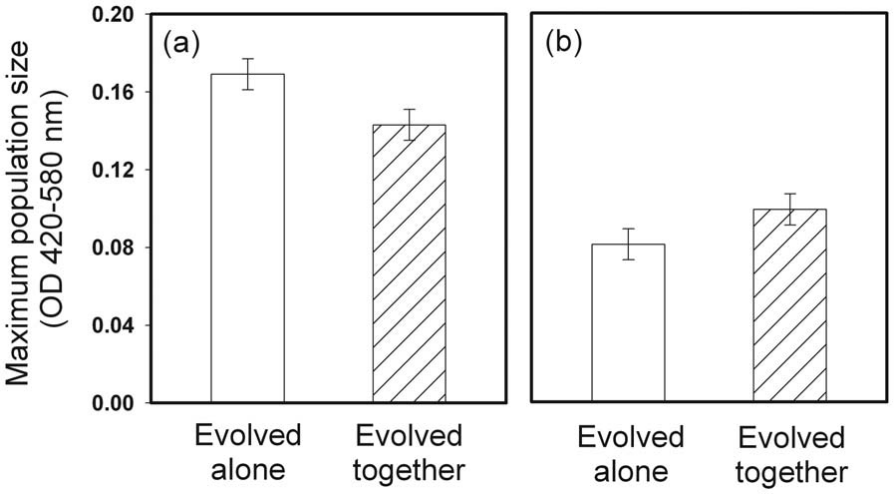
Evolutionary changes in bacterial maximum population size and resistance against ancestral PPV-phage. Maximum population densities of bacteria in the absence (panel a) and presence (panel b) of phage. Solid bars denote bacteria that evolved alone, and dashed bars bacteria that coevolved with the phage during the microcosm experiment. Phage selection increase bacterial phage-resistance at the expense of decreased maximum population size in the absence of phage. The data is pooled over temperature treatments and the error bars denote ± s.e.m. (N = 5).

We found that phage selection increased the bacterial maximum population size in the presence of phages, but decreased bacterial maximum population size in the absence of phages (a significant interaction between the presence of phage in the past and presence of phage in the phage-resistance measurements (F_1, 292_ = 8.03, *P* = 0.005, Fig. 1)). A similar, but non-significant, trend was also found with maximum growth rate of bacteria (data not shown, F_1, 288_ = 3.74, *P* = 0.054): evolution of phage-resistance tended to decrease the growth rate of bacteria in the absence of phages.

Temperature and phage selection had non-significant main effects on bacterial growth traits (*P*>0.1 in all cases), while the high measurement temperature (37°C) used in the trait measurements increased the maximum growth rate of bacteria by 37% in general (F_1, 38_ = 28.12, *P*<0.001).

Phage and temperature selection did not affect bacterial biofilm formation (all *P*>0.05), while high temperature (37°C) that was used in the trait measurements increased bacterial biofilm formation by 23% in general (F_1, 38_ = 200.77, *P*<0.001).

**Figure 2:**
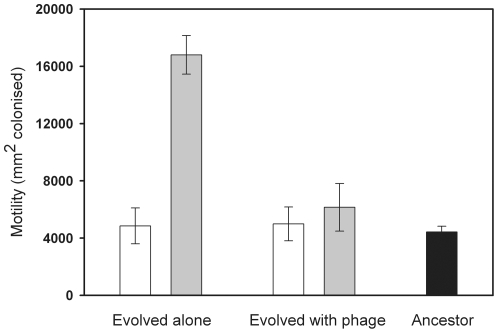
Evolution of bacterial motility. Motility of *S. marcescens* bacteria that evolved in the absence or presence of PPV-phage at 25°C (white bars) and 37°C (grey bars) temperature regimes during the microcosm experiment. Black bar denotes motility of the ancestral strain. Selection due to high temperature increases bacterial motility but only in the absence of phage. All error bars denote ± s.e.m. (N = 5).

Selection at high temperature (37°C) increased bacterial motility (main effect of temperature, F_1, 76_ = 41.09 *P*<0.001). In addition, the temperature and phage treatments had interactive effects on bacterial motility (F_1, 76_ = 8.26, *P* = 0.005, Fig. 2): high temperature selection increased bacterial motility only in the absence of phages (Fig. 2). The high measurement temperature used in the trait measurements decreased bacterial motility (F_1, 76_ = 33.9, *P*<0.001, data not shown).

**Figure 3:**
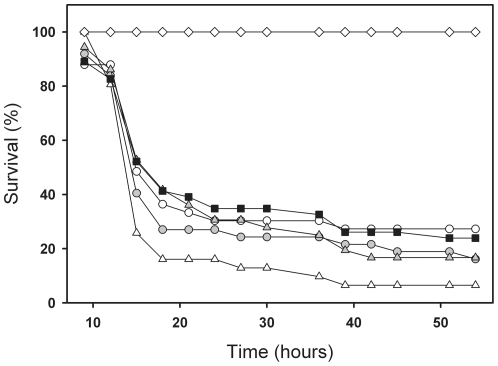
The survival of host larvae infected with bacterial strains differing their evolutionary histories. Survival of host larvae when infected with bacteria that evolved in the absence (open symbols) or presence (grey symbols) of PPV-phage in a 25°C (circles) or 37°C (triangles) during the microcosm experiment. Black squares denote the ancestral *S. marcescens* strain and diamonds the water controls. Selection due to high temperature increases bacterial virulence but only in the absence of phage.

Neither temperature or phage selection had significant main effects on bacterial pathogenicity, i.e. larval survival (Chi-Square = 0.08, *P* = 0.77 and Chi-Square = 1.42, *P* = 0.23, respectively, Fig. 3). However, we found a significant interaction between these factors, such that the past selection due to high temperature (37°C) increased bacterial pathogenicity, but only in the absence of phages (Chi-Square = 4.56, *P* = 0.033, Fig. 3).

## Discussion

In this study, we investigated how lytic bacteriophage and environmental temperature affect the evolution of bacterial pathogenicity traits *in vitro*, and how these changes correlate with bacterial virulence *in vivo*. We found that phage induced selection increased bacterial phage-resistance in general and this was costly in terms of reduced maximum population size in the absence of phages (Fig. 1). High temperature (37°C) selection increased the motility (Fig. 2) and pathogenicity of *S. marcescens* when the bacterium was cultured in the absence of phage (Fig. 3). However, past selection due to high temperature did not affect bacterial pathogenicity in the presence of the parasitic PPV-phage (Fig. 3). These results suggest that high environmental temperature could select for more virulent bacteria in environmental reservoirs, while selection by phages could constrain this effect.

High temperature selection had no effect on bacterial maximum growth rate and biofilm formation, even though using 37°C temperature in bacterial trait measurements increased the mean values of these traits in general. However, high temperature selection increased *S. marcescens* motility in the absence of phages (Fig. 2). It is known that complex regulatory networks link motility and bacterial pathogenicity. Motility can, for example, help pathogens colonise suitable niches within the host [23], [24], [41]. Although the motility of *S. marcescens* has previously been observed to decrease at 37°C [49], a recent study has demonstrated that a mutation in the *rssA* gene can increase the swarming motility of *S. marcescens* relative to the ancestral strain, at both 30°C and 37°C [50]. Our results thus suggest that a temperature-controlled increase in *S. marcescens* motility likely increased its pathogenicity in this experiment. The motility of *S. marcescens* is also known to coregulate with production of a bacterial toxin, hemolysin, which important virulence factor in *S. marcescens* [50]. Thus, it is possible that a temperature-controlled increase in *S. marcescens* pathogenicity was accompanied by a change in some other unmeasured virulence trait, such as hemolysin production.

Bacteriophages could have constrained the high temperature mediated increase of bacterial pathogenicity through at least two different mechanisms. It is known that phage-resistance often incurs a competitive growth cost [13], [17]–[19], which can result from the loss of susceptible phage attachment receptor that is also used for the uptake of nutrients [17], [18]. As a result, decrease in bacterial growth could lead to less efficient host exploitation during the infection and thus decrease bacterial pathogenicity [4], [25], [26]. According to trait measurements, bacteria evolved higher resistance against ancestral phages during the microcosm experiment (Fig. 1, panel b) and that phage-resistance was costly in terms of reduced bacterial maximum population size in the absence of phages (Fig. 1, panel a). However, temperature selection did not affect the strength of phage-resistance or the magnitude of its associated growth cost. Further, phage selection in 25°C temperature did not decrease bacterial pathogenicity relative to the ancestor or control strain (Fig. 3), and the phage-resistance did not trade-off with bacterial growth rate, which is expected to ultimately define how fast pathogen can exploit its host [25]. These results suggest that the phage induced growth cost was of a small magnitude and thus unlikely constrained the temperature-induced increase in bacterial pathogenicity.

The increase in bacterial pathogenicity was most clearly accompanied with increase in motility, while high temperature selection did not increase bacterial motility in the presence of bacteriophages (Fig. 2). Low motility has often been connected to resistance against phages [20]–[22]. This could be due to the fact that many parasitic phages actually use the bacterial pilus or flagella (including *S. marcescens*) as the receptor for their attachment [22], [41]–[43]. If this is also the case with our phage, it is possible that the temperature-controlled increase in bacterial motility (and flagellin expression) could have increased the probability to get infected by phages [41], [42]. Alternatively, it is possible that high motility could directly increase bacterial mortality by leading to more frequent encounters with phages. For example, the mixing of bacteria and phage populations can speed up the rate of co-evolution by increasing the bacteria and phage encounter rates [15]. As a result, temperature-induced increase in bacterial motility could have been maladaptive in the presence of parasitic phages, if the phages encountered and infected the more motile bacteria more often. As a result, selection against highly motility bacteria by phages is likely to explain why temperature-induced increase in bacterial pathogenicity was observed only in the absence of phages in our experiment.

Despite the successes of modern medicine, at any time over 1.4 million people worldwide suffer from infections caused by hospital-acquired bacteria [51]. In addition, bacterial pathogens cause considerable economic costs for societies for example by causing disease outbreaks in fish farms [52] and other monocultures [53]. Thus, it is very important to improve our understanding of factors that govern the evolution of bacterial pathogenicity [51]. Our work shows that a high temperature (37°C) is an important factor in indirectly selecting for higher pathogenicity of *S. marcescens* bacteria. However, we have also shown that the magnitude of this effect could rely heavily on other environmental factors, such as the presence of bacteriophages. These results are similar to a previous study, where predation by the protozoan *Tetrahymena thermophila* led to an evolutionary decrease in the pathogenicity of *S. marcescens* bacterium [40]. Interestingly, lowered pathogenicity was accompanied by a decrease in bacterial motility [40] and resource use efficiency [40], [54]. Thus, antagonistic interactions between bacteria and either their phage or their protozoan predators could select for less pathogenic bacteria through trade-offs between defensive and virulence traits. These results contradict previous studies in which defensive traits against protozoan predators have been shown to correlate positively with the bacterial pathogenicity [6], [7], [9]–[11]. One explanation for this discrepancy could be that the effect of phages and protozoan predators on bacterial pathogenicity could depend ultimately on which study species are used. In the future it would be interesting to classify several opportunistic bacterial species and their enemies according their ecology (e.g. natural habitat) or phylogeny to test if certain ecological communities or species groups respond similarly to given selection pressures. Furthermore, comparison of clinical and natural isolates of opportunistic bacteria could reveal which trait changes are important for the shift from benign to pathogenic lifestyle.

## Materials and Methods

### Isolation of a novel bacteriophage for S. marcescens

Several bacteriophages were isolated from samples taken at a sewage plant (Jyväskylä, Finland) by the enrichment method as described below. The collected samples were transferred to 500 ml Erlenmeyer flasks, each containing 100 ml of fresh Luria Broth (LB) [55]. The flasks were inoculated with 200 µl of an overnight-grown *S. marcescens* culture (ATCC strain #13880) and maintained at room temperature for 24 hours with constant agitation of 220 rpm. Bacteria were collected by centrifugation (3 min, 13000 rpm) and 100 µl of the supernatant was spread on a Petri dish with 200 µl of host bacteria and LB-agar (0.7%). After 18 hours, plaques were observed on the bacterial lawn. A single plaque from each plate was selected and plated with the host to establish a genetically homogenous virus population. One of these bacteriophage isolates, designated as PPV, was selected for the microcosm experiment and was thus subjected to further analysis.

### Characterization of the PPV bacteriophage

Optimal conditions for the one-step growth of PPV were determined. After cultivation, the cells were collected by centrifugation (Sorvall GSA, 8000 rpm, 20 mins, 5°C). Virus particles in the supernatant were precipitated with PEG (0,5 M NaCl, 10% PEG 6000) as described in [56], and further purified in a caesium chloride density gradient (1.5 g/ml CsCl, 50 mM Tris-HCl pH 7.2, 100 mM MgCl2, 150 mM NaCl). Purified particles were stained with 1% phosphotungstic acid (PTA), pH 6.5 on carbon-coated grids, and examined with a transmission electron microscope (Jeol JEM-1200EX, 60 kV). The genomic DNA of PPV was isolated using the protocol described by [57]. This purified DNA was digested with restriction enzymes and the resulting bands were observed using agarose gel electrophoresis. Three separate restriction fragments of the genomic DNA of PPV were cloned into the pSU18 plasmid. The inserts were sequenced with ABI Prism® 3130x1. We followed the manufacturer's protocol by using the BigDye Terminator v3.1 Cycle Sequencing Kit (Applied Biosystems).

The PPV phage was determined to be a lytic virus with a head-tail morphology. The head was 55 nm in diameter and the tail was ∼10 nm long. The genome of PPV is a circular, double-stranded DNA molecule, ∼40 kbp in size. Fragments of the genomic DNA of PPV were sequenced and the sequences were BLASTed. The best matches were to RNA polymerase gene in bacteriophage T7, the tail tubular protein B gene in T7 and EcoB & EcoK restriction enzyme inhibiting genes in T7. The overall DNA identity between the sequenced fragments of PPV and T7 genomes was ∼80%. Given the sequential and morphological similarities, PPV was assigned to the T7-like bacteriophages of the *Podoviridae* family.

### Microcosm experiment

The experiment was conducted in 250 ml plastic Erlenmeyer flasks (Corning), capped with membrane filters to maintain aerobic conditions. At the beginning of the experiment, a single clone of *Serratia marcescens* (ATCC strain #13880, capable of producing red pigment prodigiosin) was grown to late log phase (approximately 3.9*10^7^ cells ml^−1^) in a sterilized bacterial culture medium (phosphate-buffered hay extract containing 2.15 mg l^−1^ final concentration of plant detritus [54]. Ten microcosms were established in both the low (25°C) and high (37°C) temperature regime. Half of the microcosms, in both temperatures, were seeded with phages as follows: 30 ml of a bacterial culture medium containing *S. marcescens* was measured into the microcosms and then approximately 10^9^ particles of the PPV-phage (75 µl) were introduced. Phage density was determined as plague forming units (PFU) on ancestral *S. marcescens* bacterial lawn. The remaining five microcosms, which contained only *S. marcescens*, were retained as control treatments in both temperature regimes. Thus, the microcosm experiment consisted of four different treatments, each with five replicates (total of 20 microcosms). The microcosms were serially renewed at four-day intervals by transferring 3 ml aliquots to new microcosms containing 27 ml of fresh bacterial culture medium. There were six renewals during the 24-day experiment (equal to approximately 720 bacterial generations).

### Isolation of bacterial clones and preparations for trait measurements

The evolutionary changes in bacterial traits were measured in a separate, short-term factorial experiment, at the end of the long-term evolutionary experiment. All bacteria were grown separately for 142 hours (6 days) before being assessed for evolutionary changes as described below. This time is equivalent to tens of bacterial generations before the trait measurements. During this time, the possible treatment-induced differences in the physiological state of study organisms is likely to reset and the observed differences can be considered to be caused by genetic factors. It is possible that the 142-hour growth period with neutral selective conditions could lead to some unwanted genetic changes. However, the fact that we observed evolutionary differences between different treatments proves that selection under this growth period was not strong enough to eradicate genetic differences driven by experimental treatments used in the long-term microcosm experiment (see Results). The bacteria were separated from the phages as described below by a method modified from [16]. 100 µl subsamples from all microcosms were mixed with 5 ml of bacterial culture medium and 0.0056% Virkon (a commercially available disinfectant). After 22 h, samples were spread on agar plates containing 2.5 g of yeast extract, 10 g of nutrient broth and 15 g of agar in 1 l of dH_2_O. The removal of phages was verified by plating; the phage removal was determined successful when no phage plaques were observed on bacterial lawn. The Virkon treatment was also applied to the ancestral strain. After 48 hours of incubation on plates at 25°C, 10 clones per microcosm were selected at random and transferred to Honeycomb 2 spectrophotometer plates (Oy Growth Curves Ab Ltd.), containing 400 µl of fresh bacterial culture medium. After 48 h incubation in liquid culture medium, the bacterial mixes for the infection experiment and trait measurements were prepared as follows. All clones belonging to one treatment were mixed together by pipetting 100 µl of the bacterial solution to a 15 ml centrifuge tube (VWR). 120 µl of the mixed bacterial solutions were spread on agar plates and incubated 24 h at 25°C. Next the bacterial mass was streaked and mixed with water until 2.0 of optical density at 420–580 nm (approximately 5.5*10^8^ bacterial cells ml^−1^) was reached. Finally, 0.7 ml of this bacterial solution was mixed with 0.5 ml glycerol and the bacterial strains were frozen at −80°C.

### Measuring the evolutionary changes in bacterial traits

Before the trait measurements, aliquots of each frozen bacterial strain were thawed for 1 h at 25°C. All bacterial traits were measured for both 25°C and 37°C temperatures, regardless of their evolutionary history.

To assess changes in the bacterial resource use efficiency, small bacterial inoculums from each microcosm (<0.0002% of the maximum population size) were added to a fresh bacterial culture media. We determined the maximum population size and maximum growth rate from the biomass growth data at 10 min intervals for 96 hours (420–580 nm optical density). In our experimental setting, these traits reflects competitive ability at the time when the bacteria were serially transferred to the new microcosms with lots of fresh resources (‘maximum growth rate’ trait), and when the resources became well consumed at the end of the 4-day long serial transfer schedule (‘maximum population size’). Both of these growth traits are potentially related to bacterial fitness and could be traded off with phage-resistance.

Phage-resistance was measured as bacterial growth in the presence of ancestral phages. Small inoculums of bacteria (10 µl of bacteria at the late log phase) and the ancestral phage (10 µl inocula containing ∼10^7^ phage particles determined as PFU count) were added simultaneously to a fresh bacterial culture media (400 µl). Phage-resistance was measured as the maximum population size and maximum growth rate of the bacteria in the presence of phages using the biomass growth data recorded for 96 hours at 10 min intervals (420–580 nm optical density). Even though the ancestral phage stock was washed with water two times prior to the measurements (by using the Amicon Ultra Centrifugal Filter Device 10 000, Millipore), the phage inoculums still contained some nutrients, which increased bacterial growth. Therefore, similar inoculums of heat-inactivated phages (15 min in 100°C water bath) were added to the bacteria-alone treatments to control for this effect (bacterial growth in the absence of phages).

The bacterial motility assay was conducted by stabbing a trace amount (2 µl) of each bacterial strain onto the centre of a semi-fluid NB agar plate (containing 0.7% agar) with a sterile loop (VWR). The motility of strains was recorded as the area (mm^2^) of the agar plate the bacteria were able to colonise in 96 h. Motility measurements were replicated twice for all the experimental treatments and six times for the ancestral strain).

Bacterial ability to form biofilm was measured by using a method modified from [58]. After four days of bacterial growth in 400 µl of fresh bacterial culture media, 100 µl of 1% crystal violet solution (Sigma-Aldrich) was added to each well of a microtitre plate (Honeycomb 2, Thermo Electron Oy). After 10 minutes, all wells were rinsed with distilled water three times and 450 µl of 96% ethanol was added to all wells, to dissolve the crystal-violet stained bacteria from the walls of wells. The amount of biofilm was estimated by measuring the optical density of the crystal violet/ethanol solution for 24 h at 420–580 nm.

### Infection experiment and the measurement of bacterial pathogenicity

Wood tiger moth larvae (*Parasemia plantaginis*, Arctiidae) were used as insect model hosts, to determine changes in bacterial pathogenicity [40]. The larvae were from lab stock and had been reared under controlled conditions: temperature and rearing density were kept constant, while fresh food (dandelion, *Taraxacum* sp.) was offered *ad libitum* [40]. Infection was performed by the injection method (needle). This technique bears a resemblance to the infection by pathogenic bacteria that occasionally invade insect hemocoel directly through breaches of the cuticle [59], or on plant spines [60]. Before infection, aliquots of all bacterial strains were thawed for 1 h at 25°C. Either 5 µl of a well-mixed bacterial solution (approximately 1.66*10^6^ bacterial cells), or 5 µl of sterilized water (for the controls), were injected between the second and third segments of the larvae, with a 10 µl Hamilton syringe. The infection of larvae, and their consequent monitoring of survival was performed at 25°C. A total of 210 larvae were injected during the experiment: 27 control larvae with water, 46 larvae with the ancestral *S. marcescens* strain, 70 larvae with the *S. marcescens* strains that had evolved in the absence (N = 36) or presence (N = 34) of phages at 25°C and 67 larvae with the *S. marcescens* strains that had evolved in the absence (N = 32) or presence (N = 35) of phages at 37°C. Total of 36 larval families were used for the infection assay. To control the effect of host background, larvae from every family were divided as equally as possible between infection treatments. As a result, all infection treatments included larvae from minimum of 27 families (water control), while additional families were used to increase sample size in other infection treatments. Four microcosm replicates of each bacterial treatment group were used and injected separately to larvae (N = 8 or 9). The fifth microcosm replicates were not used due to lack of large enough larvae in the midsummer generation of *P. plantaginis*. The infection took place over 2 consecutive days under constant laboratory conditions and the larval survival was not affected by the day of infection (The Kaplan-Meier survival analysis, Log-rank statistics: Chi-Square = 1.23, *P* = 0.26). Before infection, all larvae were weighed (only larvae between 90 and 160 mg were used) and assigned to groups with approximately the same mean weight, to exclude possible condition-dependent effects (the larval weight between the bacterial treatments was not statistically significant, F_4, 182_ = 0.06, *P* = 0.99). Although bacterial pathogens might infect their hosts most frequently through the oral route, it has been shown that injection and oral infection methods correlate positively, even though the death rate of infected bacteria is usually much slower following oral infection [61]. Most importantly, recent studies have demonstrated that invertebrate and vertebrate host models correlate very well, which suggests that insect models can reflect reliably bacterial pathogenicity [46]–[48].

### Statistical analysis

The Kaplan-Meier survival analysis and Log-rank statistics were used to analyse the survival data. The main effects of bacterial treatment and temperature were first analysed separately. To examine two-way interactions, the effects of bacterial treatment and temperature were analysed using stratification. The effect of microcosm replicates was not included into the survival analysis. The right censoring method was used to include the larvae that did not die within 54 hours. Bacterial traits were analysed with two-way ANOVA. Five measurement replicates were used for every microcosm when the phage-resistance was measured (measurement replicates nested under microcosm and microcosms nested under treatment). Otherwise, measurement replicates were not used for bacterial trait analysis. All data used in ANOVA analyses met the assumption of equal variances between treatments. The statistical analyses were performed using SPSS-software (v. 17.0, SPSS Inc., Chicago, IL).

## Supporting Information

Figure S1 **Methods diagram.**
(PDF)

## References

[1] Read AF (1994). The evolution of virulence.. Trends Microbiol.

[2] Levin BR, Svanborg Eden C (1990). Selection and the evolution of virulence in bacteria: an ecumenical excursion and modest suggestion.. Parasitology.

[3] Levin BR (1996). The evolution and maintenance of virulence in microparasites.. Emerg Infect Diseases.

[4] Harrison EF, Browning L, Vos M, Buckling A (2006). Cooperation and virulence in acute *Pseudomonas aeruginosa* infections.. BMC Biology.

[5] Inglis RF, Gardner A, Cornelis P, Buckling A (2009). Spite and virulence in the bacterium *Pseudomonas aeruginosa*.. Proc Natl Acad Sci USA.

[6] Cirillo JD, Cirillo SL, Yan L, Bermudes LE, Falkow S (1999). Intracellular growth in *Acanthamoeba castellanii* affects monocyte entry mechanisms and enhances virulence of *Legionella pneumophila*.. Infect Immun.

[7] Harb OS, Gao L-Y, Kwaik YA (2000). From protozoa to mammalian cells: a new paradigm in the life cycle of intracellular bacterial pathogens.. Environ Microbiol.

[8] Matz C, Kjelleberg S (2005). Off the hook – how bacteria survive protozoan grazing.. Trends microbiol.

[9] Rasmussen MA, Carlson SA, Franklin SK, McCuddin ZP, Wu MT (2005). Exposure to rumen protozoa leads to enhancement of pathogenicity of and invasion by multiple-antibioticresistant *Salmonella enterica* bearing SGI1.. Infect Immun.

[10] Steinberg KM, Levin BR (2007). Grazing protozoa and the evolution of the *Escherichia coli* O157:H7 Shiga toxin-encoding prophage.. Proc Roy Soc Lond B.

[11] Lainhart W, Stolfa G, Koudelka GB (2009). Shiga toxin as a bacterial defense against a eukaryotic predator, *Tetrahymena thermophila*.. J Bacteriol.

[12] Brüssow H (2007). Bacteria between protests and phages: from antipredation strategies to the evolution of pathogenicity.. Mol Microbiol.

[13] Bohannan BJM, Lenski RE (1999). Effect of prey heterogeneity on the response of a model food chain to resource enrichment.. Am Nat.

[14] Buckling A, Rainey PB (2002). Antagonistic coevolution between a bacterium and a bacteriophage.. Proc Roy Soc Lond B.

[15] Brockhurst MA, Morgan AD, Rainey PB, Buckling A (2003). Population mixing accelerates coevolution.. Ecol Lett.

[16] Morgan AD, Gandon S, Buckling A (2005). The effect of migration on local adaptation in a coevolving host-parasite system.. Nature.

[17] Lenski RE, Levin BR (1985). Constraints on the coevolution of bacteria and virulent phage: a model, some experiments, and predictions for natural communities.. Am Nat.

[18] Lenski RE (1988). Dynamics of interactions between bacteria and virulent bacteriophage.. Adv Microb Ecol.

[19] Brockhurst MA, Rainey PB, Buckling A (2004). The effect of spatial heterogeneity and parasites on the evolution of host diversity.. Proc R Soc Lond B.

[20] Heierson A, Sidén I, Kivaisi A, Boman H (1986). Bacteriophage-resistant mutants of *Bacillus thuringiensis* with decreased virulence in pupae of *Hyalophora cecropia*.. J Bacteriol.

[21] Paruchuri DK, Harshey RM (1987). Flagellar variation in *Serratia marcescens* is associated with color variation.. J Bacteriol.

[22] Brockhurst MA, Buckling A, Rainey PB (2005). The effect of a bacteriophage on diversification of the opportunistic bacterial pathogen, *Pseudomonas aeruginosa*.. Proc R Soc Lond B.

[23] Josenhans C, Suerbaum S (2002). The role of motility as a virulence factor in bacteria.. Int J Med Microbiol.

[24] Lane MC, Alteri CJ, Smith S, Mobley HLT (2007). Expression of flagella is coincident with uropathogenic *Escherichia coli* ascension to the upper urinary tract.. Proc Natl Acad Sci USA.

[25] Frank SA (1996). Models of parasite virulence.. Q Rev Biol.

[26] de Roode JC, Pansini R, Cheesman SJ, Helinski MEH, Hujben S (2005). Virulence and competitive ability in genetically diverse malaria infections.. Proc Natl Acad Sci USA.

[27] Flyg C, Kenne K, Boman HG (1980). Insect pathogenic properties of *Serratia marcescens*: phageresistant mutants with a decreased resistance to *Cecropia* immunity and a decreased virulence to *drosophila*.. J Gen Microbiol.

[28] Santader J, Robeson J (2007). Phage-resistance of *Salmonella enterica* serovar Enteritidis and pathogenesis in *Caenorhabditis elegans* is mediated by the lipopolysaccharide.. Electron J Biotechnol.

[29] Duriez P, Zhang Y, Lu Z, Scott A, Topp E (2008). Loss of virulence genes in *Escherichia coli* populations during manure storage on a commercial swine farm.. Appl Environ Microbiol.

[30] Ellis CN, Cooper VS (2010). Experimental adaptation of *Burkholderia cenocepacia* to onion medium reduces host range.. Appl Environ Microbiol.

[31] Konkel ME, Tilly K (2000). Temperature-regulated expression of bacterial virulence genes.. Microb Infect.

[32] Maurelli AT, Blackmon B, Curtis R, III (1984). Temperature-dependent expression of virulence genes in Shigella species.. Infect Immun.

[33] Mauchline WS, James BW, Fitzgeorge RB, Dennis PJ, Keevil CW (1994). Growth temperature reversibly modulates the virulence of *Legionella pneumophila*.. Infect Immun.

[34] Pulkkinen K, Suomalainen L-R, Read AF, Ebert D, Rintamäki P (2010). Intensive fish farming and the evolution of pathogen virulence: the case of columnaris disease in Finland.. Proc Roy Soc Lond B.

[35] Case RJ, Longford SR, Cambell AH, Low A, Tujula N (2010). Temperature induced bacterial virulence and bleaching disease in a chemically defended marine macroalga..

[36] Bennett AF, Dao KM, Lenski RE (1990). Rapid evolution in response to high temperature selection.. Nature.

[37] Bennett AF, Lenski RE, Mittler JE (1992). Evolutionary adaptation to temperature. I. Fitness responses of *Escherichia coli* to changes in its thermal environment.. Evolution.

[38] Wei J-R, Lai H-C (2006). N-Acylhomoserine lactone-dependent cell-to-cell communication and social behavior in the genus *Serratia*.. Int J Med Microbiol.

[39] Grimont PAD, Grimont F (1978). The genus Serratia.. Annu Rev Microbiol.

[40] Friman V-P, Lindstedt C, Hiltunen T, Laakso J, Mappes J (2009). Predation on multiple trophic levels shapes the evolution of pathogen virulence.. PLoS ONE.

[41] Malik-Kale P, Raphael BH, Parker CT, Joens LA, Klena JD Characterization of genetically matched isolates of Campylobacter jejuni reveals that mutations in genes involved in flagellar biosynthesis alter the organism's virulence potential.. Appl Environ Microbiol.

[42] Meynell EW (1961). A phage, Φx, which attacks motile bacteria.. J Gen Microbiol.

[43] Iino T, Mitani M (1967). Infection of *Serratia marcescens* by Bacteriophage x.. J Virol.

[44] Samuel ADT, Pitta TP, Ryu WS, Danese PN, Leung ECW (1999). Flagellar determinants of bacterial sensitivity to x-phage.. Proc Natl Acad Sci USA.

[45] Donlan RM (2009). Preventing biofilms of clinically relevant organisms using bacteriophage.. Trends Microbiol.

[46] Jander G, Rahme LG, Ausubel FM (2000). Positive correlation between virulence of *Pseudomonas aeruginosa* mutants in mice and insects.. J Bacteriol.

[47] Brennan M, Thomas DY, Whiteway M, Kavanagh K (2002). Correlation between virulence of *Candida albicans* mutants in mice and *Galleria mellonella* larvae.. FEMS Immunol Med Microbiol.

[48] Mukherjee K, Altincicek B, Hain T, Domann E, Altincicek A (2010). *Galleria mellonella* as a model system for studying *Listeria* pathogenesis.. _Appl Environ Microbiol.

[49] Liu JH, Lai MJ, Ang S, Shu JC, Soo PC (2000). Role of *flhDC* in the expression of the nuclease gene *nucA*, cell division and flagellar synthesis in *Serratia marcescens*.. J Biomed Sci.

[50] Lai HC, Soo PC, Wei JR, Yi WC, Liaw SJ (2005). The RssAB two-component signal transduction system in *Serratia marcescens* regulates swarming motility and cell envelope architecture in response to exogenous saturated fatty acids.. J Bacteriol.

[51] Ducel G, Fabry J, Nicolle L (2002). Prevention of hospital-acquired infections: *A* PRACTICAL GUIDE..

[52] Austin B, Austin DA (2007). Bacterial Fish Pathogens: Disease of Farmed and Wild Fish..

[53] Mew TW (1987). Current status and future prospects of research on bacterial blight of rice.. Ann Rev Phytopathol.

[54] Friman V-P, Hiltunen T, Laakso J, Kaitala V (2008). Prey resource availability drives evolution of predator prey interaction.. Proc R Soc Lond B.

[55] Sambrook J, Russel DW (2001). Molecular Cloning: a Laboratory Manual..

[56] Hinnen R, Schäfer R, Franklin RM (1974). Structure and synthesis of lipid-containing bacteriophage. Preparation of virus and localization of the structural proteins.. Eur J Biochem.

[57] Bamford JKH, Bamford DH (1991). Large-scale purification of membrane-containing bacteriophage PRD1 and its subviral particles.. Virology.

[58] O'Toole GA, Kolter R (1998). The initiation of biofilm formation in *Pseudomonas aeruginosa* WCS365 proceeds via multiple, convergent signaling pathways: a genetic analysis.. Mol Microbiol.

[59] Vallet-Gely I, Lemaitre B, Boccard F (2008). Bacterial strategies to overcome insect defences.. Nature Rev Microbiol.

[60] Halpern M, Raats D, Lev-Yadun S (2007). Plant biological warfare: Thorns inject pathogenic bacteria into herbivores.. Env Microbiol.

[61] Nehme NT, Liégeois S, Kele B, Giammarinaro P, Pradel E A model of bacterial intestinal infections in *Drosophila melanogaster*.. PLoS Pathog.

