# Supplementary material for: High Temperature and Bacteriophages Can Indirectly Select for Bacterial Pathogenicity in Environmental Reservoirs
Source: PLoS One. 2011 Mar 15;6(3):e17651. doi: 10.1371/journal.pone.0017651 (PMC3057980; doi:10.1371/journal.pone.0017651)
Supplement: Figure S1 — Methods diagram. (PDF) [file pone.0017651.s001.pdf]

## Selection experiment for 24 days, 90% resource renewal every 4th day

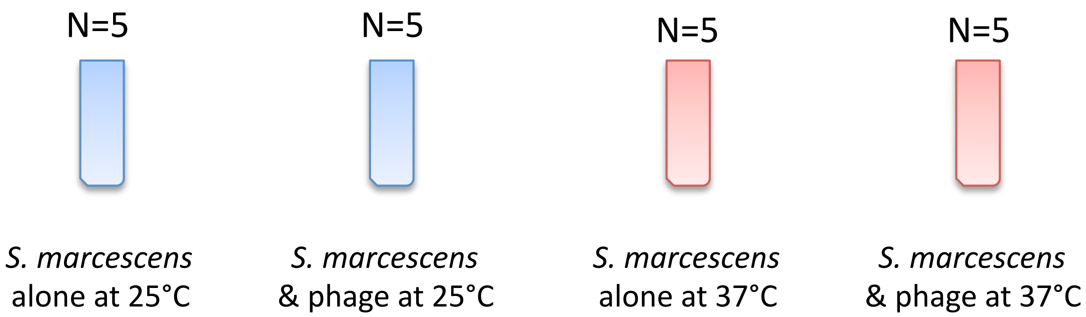


## Preparing bacteria for the trait measurements:

1) Virkon treatment to kill phage
2) Dilution and plating of clones
3) Growth of isolated clones in separation
4) Preparation of clone mixes and measuring evolutionary changes

## Bacterial trait measurements:

| At 25°C | At 37°C |
|---|---|
| Phage-resistance | Phage-resistance |
| Maximum population size | Maximum population size |
| Maximum growth rate | Maximum growth rate |
| Biofilm formation | Biofilm formation |
| Motility | Motility |
| Virulence *in vivo* | - |
